# Reproducible and scalable differentiation of highly pure cortical neurons from human induced pluripotent stem cells

**DOI:** 10.1016/j.xpro.2023.102266

**Published:** 2023-05-04

**Authors:** Angelika Dannert, Julien Klimmt, Carolina Cardoso Gonçalves, Dennis Crusius, Dominik Paquet

**Affiliations:** 1 Institute for Stroke and Dementia Research (ISD), University Hospital, LMU Munich, 81377 Munich, Germany; 2 Graduate School of Systemic Neurosciences, LMU Munich, 81377 Munich, Germany; 3 Munich Cluster for Systems Neurology (SyNergy), 81377 Munich, Germany

**Keywords:** Cell Biology, Cell culture, Neuroscience, Stem Cells, Cell Differentiation

## Abstract

Human-induced-pluripotent-stem-cell (hiPSC)-derived neurons are valuable for investigating brain physiology and disease. Here, we present a protocol to differentiate hiPSCs into cortical neurons with high yield and purity. We describe neural induction via dual-SMAD inhibition, followed by spot-based differentiation to provide high quantities of neural precursors. We detail their enrichment, expansion, and purification to avoid unwanted cell fates and provide optimal conditions for neural rosette proliferation. These differentiated neurons are suitable for drug testing and co-culture studies.

For complete details on the use and execution of this protocol, please refer to Paquet et al.^1^ and Weisheit et al..^2^

## Highlights

• Generation of highly pure hiPSC-derived cortical neurons by spot-based differentiation
• Expansion and purification steps to avoid proliferative cell/astrocyte contaminations
• Neuronal precursor cells (NPCs) can be cryopreserved for cell banking
• Stable long-term culture of differentiated cortical neurons for up to one year

## Abstract (editor highlights)

Publisher’s note: Undertaking any experimental protocol requires adherence to local institutional guidelines for laboratory safety and ethics.

## Graphical abstract

**Figure:**
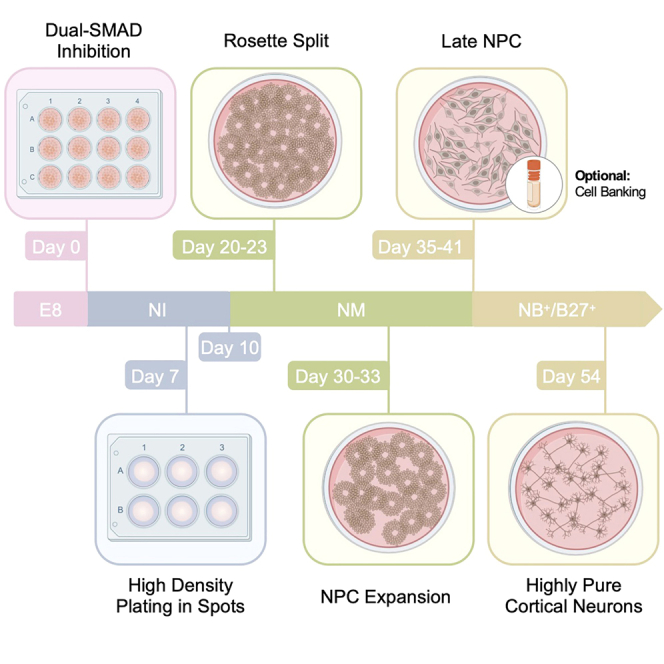

## Before you begin

### Institutional permissions

Experiments on pluripotent stem cells need to be performed in accordance with relevant institutional and national guidelines and regulations and may need to be approved by an institutional review board (IRB) or similar committee. This was also performed for the experiments described in this study, where applicable.

### Culture of human induced pluripotent stem cells (iPSCs)

**Timing: around 1 week prior to starting the differentiation, with one iPSC maintenance split performed before starting the differentiation**

1. Thawing of induced pluripotent stem cells (iPSCs)
  a. Prepare vitronectin-coated plates: add 1 mL of vitronectin solution (1:100 in PBS) for each well of a 6-well plate, coat for at least 1 h at room temperature (RT – 20°C–22°C)
  b. Prepare 9 mL DMEM-F12 and 2 mL E8 Flex/RI (RI = Rock-inhibitor Y27632, 10 μM) for each thawed tube, warm both to RT.
  c. Get stored cryovials with iPSCs from liquid nitrogen tank and thaw in a 37°C water bath until only a small ice clump remains.
  d. Slowly add 1 mL of prepared DMEM-F12 to each cryovial to dilute DMSO using a sterile Pasteur pipette.
  e. Quickly transfer the thawed cell solution to the prepared 15 mL conical tube containing DMEM-F12, avoid generating single cells by trituration.
  f. Spin at 170 *g* for 4 min.
    ***Note:*** all centrifugation steps are performed with these parameters at RT unless indicated otherwise
  g. Aspirate supernatant, resuspend cells in 2 mL of E8 Flex/RI, remove vitronectin solution from the prepared plate and carefully plate cell solution into one well of the 6-well plate.
  h. Change media as needed (feed with E8 Flex without RI), split cells after 2–3 days.
    ***Note:*** iPSCs in E8 Flex can be kept without a media change for up to 3 days, as long as the color of the medium is not changing to yellow.
    ***Alternatives:*** Alternative media can be used for iPSC maintenance; we successfully tested mTESR, StemFlex, and E8 with our neuron differentiation protocol; when using other media (in particular those not based on heat-stabilized bFGF) the feeding schedule needs to be adjusted accordingly, and we refer the reader to the respective manufacturer’s instructions.

2. Culture of iPSCs.
  a. iPSC lines growing in E8 Flex are typically split ∼1:6 every 3–4 days (e.g., Tuesday and Friday) and are kept in colonies. Before splitting, cells should form dense colonies with a smooth and uniform surface.
  b. Split cultures when (or preferentially before) the first of the following occurs:
    i. iPSC colonies become too dense (cells start to pile up) or too large.
    ii. iPSC colonies show increased differentiation.
    iii. iPSC colonies cover ∼80% of the surface area of the culture vessel, usually every 3–4 days.
3. Splitting iPSC colonies:
  a. Prepare and warm E8 Flex medium and DMEM-F12 to RT as needed, coat wells of a 6-well plate with vitronectin as needed
  b. Add 1.5 mL E8 Flex per well to recipient, vitronectin-coated plate (no RI is used for normal splitting).
    ***Note:*** If iPSCs are split on a Friday, 3.5 mL can be added to skip feeding on the weekend.
  c. If spontaneously differentiated cells are present, remove them from the plate using a dissection microscope, or by aspirating non-translucent areas/colonies on culture plates (see “Critical” paragraph below).
  d. Aspirate culture medium, wash cells with 1 mL PBS.
  e. Remove PBS, add 1 mL PBS with 0.5 μM EDTA and incubate 4–5 min at RT, monitor cells every 1–2 min to check morphology, cells are ready to split when colonies develop gaps in the middle but are still loosely attached.
  f. Aspirate PBS/EDTA without dislodging colonies.
  g. Forcefully add DMEM-F12 (half the volume of the split ratio, i.e., for a 1:6 split, add 3 mL) to the well using a sterile plastic Pasteur pipette to wash off colonies. This works best by pushing the DMEM-F12 ‘under the colonies’ from the side.
  h. Pull up media, and push out forcefully to wash residual colonies off the plate, pipette up and down until small colonies remain (usually 2–5 times).
    ***Note:*** iPSCs should always be kept in colonies to avoid cell death or spontaneous differentiation of single cells.
  i. Avoid scraping the cells from the culture vessel during passaging, as differentiated cells preferentially stay attached.
  j. Quickly add 0.5 mL of cell suspension into each new well containing fresh E8 Flex media (prepared in step 3b) using the same Pasteur pipette before the clumps start to settle down in the pipette.
  k. Evenly distribute colonies by moving the plate back and forth in two directions.
  l. No feed is required for the next 2 days, do 2 mL feeds on following days until the next split.
    ***Note:*** If using another iPSCs media (not E8 Flex) which does not contain heat-stabilized bFGF, timing of the feeds needs to be adjusted accordingly, i.e., feed every day or every other day.
    **CRITICAL:** After thawing, iPSCs should be passaged at least once prior to starting the differentiation protocol. It is critical that aberrant differentiation of iPSCs is below 5% before the neural induction is started. If necessary, remove spontaneously differentiated cells from the iPSC cultures as per standard stem cell culture procedures (e.g., manual removal under a microscope or reduced incubation time of 4 min with PBS/EDTA, as iPSCs usually detach before differentiated cells).

## Key resources table

**Table:** 

REAGENT or RESOURCE	SOURCE	IDENTIFIER
**Antibodies**		

CTIP2 (1:300)	Abcam	ab18465
FoxG1 (1:200)	Abcam	ab18259
GAD67 (1:500)	Millipore	MAB5406
GFAP (1:500)	Synaptic Systems	173004
K9JA (Tau) (1:1000)	Dako	A0024
MAP2 (1:1000)	Millipore	AB15452
NANOG (1:500)	Cell Signaling	4903
Nestin (1:500)	Millipore	MAB5326
OCT4 (1:500)	Stemgent	S090023
Pax6 (1:500)	BioLegend	901302
PSD95 (1:100)	Thermo Fisher	MA1-046
S100β (1:500)	Sigma-Aldrich	S2532
SATB2 (1:100)	Abcam	ab51502
SSEA4 (1:500)	Abcam	ab16287
Synapsin-1 (1:500)	Cell Signaling	5297
Tbr1 (1:500)	Millipore	AB2261
TRA-1-60 (1:500)	Millipore	MAB4360_2016625
TuJ1 (β3-Tubulin) (1:500)	Covance	MRB-435P
vGlut1 (1:100)	Synaptic Systems	135302

**Chemicals, peptides, and recombinant proteins**		

2-Mercapto-ethanol	Thermo Fisher	21985-023
5-Fluorouracil	Sigma-Aldrich	F6627
Accutase	Sigma-Aldrich	A6964-100ML
B27 supplement (50×, with Vit. A)	Thermo Fisher	17504044
B27PLUS (50×)	Thermo Fisher	A3582801
bFGF	STEMCELL Technologies	78003.2
DAPT	Selleckchem	S2215
DMEM/F-12	Thermo Fisher	11320-074
DMSO	Sigma-Aldrich	D2650
DPBS, no calcium, no magnesium	Invitrogen	14190094
EDTA (0.5 M, pH 8,0)	Thermo Fisher	15575020
Essential 8 Flex medium	Thermo Fisher	A2858501
Fluo-4 AM	Biomol	ABD-20552
Geltrex	Thermo Fisher	A1413302
GlutaMAX	Thermo Fisher	35050
HEPES solution	Sigma-Aldrich	H0887
Insulin	Sigma-Aldrich	I0516
Insulin-Transferrin-Selen (ITS-G)	Thermo Fisher	41400-045
L-Ascorbic acid 2-phosphate sesquimagnesium salt hydrate	Sigma-Aldrich	A8960
Laminin	Thermo Fisher	23017015
LDN-193189	Selleckchem	S2618
N-2 supplement (100×)	Thermo Fisher	17502048
NEAA	Thermo Fisher	11140-050
Neurobasal	Thermo Fisher	21103-049
Neurobasal Plus	Thermo Fisher	A3582901
Penicillin-streptomycin	Thermo Fisher	15140-122
Poly-L-ornithine	Sigma-Aldrich	P4957
PowerTrack™ SYBR Green Mastermix	Thermo Fisher	A46109
RevitaCell Supplement (100×)	Thermo Fisher	A2644501
Rock Inhibitor Y27632	Selleckchem	S1049
SB-431542	Selleckchem	S1067
StemDiff™ Neural Rosette Selection Reagent	STEMCELL Technologies	5832
UltraPure™ DNase/RNase-Free Distilled Water	Thermo Fisher	10977035
Uridine	Sigma-Aldrich	U3750
Vitronectin	Thermo Fisher	A14700

**Critical commercial assays**		

High-Capacity cDNA Reverse Transcription Kit	Thermo Fisher	4368814
NucleoSpin RNA/Protein kit	Macherey Nagel	740933250

**Experimental models: Cell lines**		

7889SA2 (use for max. 10 passages)	Published (Paquet et al., 2016)^1^	N/A
A18944 (use for max. 10 passages)	Thermo Fisher	A18945
KOLF2.1 (use for max. 10 passages)	Published (Pantazis et al.^3^)	N/A

**Oligonucleotides**		

Primer: RPL22 Forward	Integrated DNA Technologies (IDT)	CACGAAGGAGGAGTGACTGG
Primer: RPL22 Reverse	IDT	TGTGGCACACCACTGACATT
Primer: GFAP Forward	IDT	AGAGAGGTCAAGCCCAGGAG
Primer: GFAP Reverse	IDT	GGTCACCCACAACCCCTACT
Primer: Ki67 Forward	IDT	GAGAATCTGTGAATCTGGGTAA
Primer: Ki67 Reverse	IDT	CAGGCTTGCTGAGGGAAT
Primer: MAP2 Forward	IDT	TTGGTGCCGAGTGAGAAGA
Primer: MAP2 Reverse	IDT	GTCTGGCAGTGGTTGGTTAA
Primer: MAPT Forward	IDT	CCAAGTGTGGCTCATTAGGCA
Primer: MAPT Reverse	IDT	CCAATCTTCGACTGGACTCTGT
Primer: PSD95 Forward	IDT	TCCACTCTGACAGTGAGACCGA
Primer: PSD95 Reverse	IDT	CGTCACTGTCTCGTAGCTCAGA
Primer: SYN1 Forward	IDT	TACAACGTACCCCGTGGTTG
Primer: SYN1 Reverse	IDT	TTTGGCATCGATGAAGGGCT
Primer: Nestin Forward	IDT	TCAAGATGTCCCTCAGCCTGGA
Primer: Nestin Reverse	IDT	AAGCTGAGGGAAGTCTTGGAGC
Primer: Oct-4 Forward	IDT	GTGTTCAGCCAAAAGACCATCT
Primer: Oct-4 Reverse	IDT	GGCCTGCATGAGGGTTTCT
Primer: NANOG Forward	IDT	AGCAGATGCAAGAACTCTCCAA
Primer: NANOG Reverse	IDT	TGAGGCCTTCTGCGTCACAC
Primer: Pax6 Forward	IDT	CGAGATTTCAGAGCCCCATA
Primer: Pax6 Reverse	IDT	AAGACACCACCGAGCTGATT
Primer: NMDAR1 Forward	IDT	CCAGTCAAGAAGGTGATCTGCAC
Primer: NMDAR1 Reverse	IDT	TTCATGGTCCGTGCCAGCTTGA

**Software and algorithms**		

GraphPad Prism 9.2.0	GraphPad Software	https://www.graphpad.com/
ImageJ	Published (Schindelin et al)^4^	https://imagej.nih.gov/ij/
Primer3Plus (used for qPCR primer design)	Published (Untergasser et al.)^5^	https://www.bioinformatics.nl/cgi-bin/primer3plus/primer3plus.cgi

**Other**		

6-well plates	Corning	353046
12-well plates	Corning	3513
Dissection microscope	Nikon	SMZ1270

## Materials and equipment

### Coatings for cell culture plates

• Vitronectin.
  ○ Stock concentration: 0.5 mg/mL.
  ○ Dilute 1:100 in PBS for coating.
  ○ Incubate plates with coating solution for at least 1 h at RT.
  ○ Store coated plates at 4°C for up to 4 weeks.
• Geltrex (GTX).
  ○ Original concentration: 12–18 mg/mL.
  ○ Dilute 1:150 in DMEM-F12 for coating.
  ○ Incubate plates with coating solution for at least 1 h at 37°C (followed by 1 h at RT if possible).
  ○ Store coated plates at 4°C for up to 4 weeks.
• Poly-L-Ornithine (PO).
  ○ Stock concentration: 5 mg/mL (resuspend 100 mg in 20 mL sterile MilliQ water).
  ○ Dilute stock 1:100 in sterile MilliQ water for coating.
  ○ Incubate plates with coating solution for at least 4 h at RT.
  ○ Store coated plates at 4°C for up to 4 weeks.
• Laminin (L).
  ○ Original concentration 1 mg/mL.
  ○ Dilute 1:100 in PBS for coating.
  ○ Pre-treat plates with PO as described above.
  ○ Aspirate PO, add Laminin-coating solution and incubate plates for at least 12 h at RT.
  ○ Plates coated with PO and L are referred to as POL-coated.
  ○ Store POL-coated plates at 4°C for up to 2 weeks.
  ○ Prepare fresh plates for the high-density split into spots.

***Note:*** All coating solutions are temperature sensitive and should therefore be aliquoted on ice, diluted in cold diluent and added to the respective plates as quickly as possible.

***Alternatives:*** both Cultrex and Matrigel can be used as alternatives to GTX, according to individual manufacturer’s instructions. Adjust the dilutions accordingly to achieve the same final concentrations.

**Table:** Neuronal Differentiation Medium (NM)

Reagent	Final concentration	Amount
Neurobasal (1×)	0.5×	237 mL
DMEM/F12 (1×)	0.5×	237 mL
Penicillin (10.000 U/mL)/Streptomycin (10 mg/mL)	100 U/mL and 0.1 mg/mL	5 mL
B27 supplement (50×) (with Vit. A)	0.5×	5 mL
GlutaMAX (200 mM)	2 mM	5 mL
NEAA (100×)	1×	5 mL
N-2 supplement (100×)	0.5×	2.5 mL
Insulin (500 μg/mL)	2.5 μg/mL	2.5 mL
2-Mercaptoethanol (55 mM)	0.05 mM	455 μL
**Total**	**N/A**	**500 mL**

Sterile filter and store at 4°C for up to 3 weeks.

• Add bFGF as indicated in the protocol: final concentration of 20 ng/μL.

To prepare Insulin stock solution, dilute 5 mL Insulin in 95 mL sterile PBS, aliquot and store at –20°C up to 6 months.

**Table:** Neural Induction Medium (NI)

Reagent	Final concentration	Amount
NM (1×)	1×	50 mL
SB431542 (10 mM)	10 μM	50 μL
LDN-193189 (2.5 mM)	0.25 μM	5 μL
**Total**	**N/A**	**50 mL**

Can be stored at 4°C for up to 5 days.

**Table:** Neuronal Maintenance Medium (NB+/B27+)

Reagent	Final concentration	Amount
Neurobasal PLUS (1×)	1×	242 mL
GlutaMAX (200 mM)	0.5 mM	0.625 mL
Penicillin (10.000 U/mL)/Streptomycin (10 mg/mL)	100 U/mL and 0.1 mg/mL	2.5 mL
B27 PLUS (50×)	1×	5 mL
**Total**	**N/A**	**250 mL**

Can be stored at 4°C for up to 4 weeks

• Add DAPT as indicated in the protocol: 10 μM final concentration.
• Add 5-Fluorouracil and Uridine (5FU) as indicated in the protocol:
  ○ 5-Fluorouracil: 5 μM final concentration.
  ○ Uridine: 5 μM final concentration.

For alternative maintenance media see step 29 below.

**Table:** Freeze Medium for Neural Rosettes

Reagent	Final concentration	Amount
NM (1×)	0.9×	9 mL
DMSO (100%)	10%	1 mL
bFGF (100 μg/mL)	20 ng/mL	2 μL
**Total**	**N/A**	**10 mL**

Freeze medium should be prepared on the day of freezing.

**Table:** Thawing Medium for Neural Rosettes

Reagent	Final concentration	Amount
NM (1×)	1×	9.9 mL
bFGF (100 μg/mL)	20 ng/mL	2 μL
RevitaCell Supplement (100×)	1×	100 μL
**Total**	**N/A**	**10 mL**

Thawing medium should be prepared on the day of thawing.

**Table:** Plating Medium

Reagent	Final concentration	Amount
NB+/B27+ (1×)	1×	9.9 mL
DAPT (10 mM)	10 μM	10 μL
RevitaCell Supplement (100×)	1×	100 μL
**Total**	**N/A**	**10 mL**

Plating medium should be prepared on the day of plating.

**CRITICAL:**DMSO: handle only with gloves as it is absorbed through the skin very rapidly. 5-FU: has acute toxicity when swallowed and is suspected to be carcinogenic. Handle with care under a hood and use personal protection equipment. Immediately call a poison center if swallowed.

### Media for optional steps

**Table:** E6 medium (for optional E6-NI gradient)

Reagent	Final concentration	Amount
DMEM-F12 (1×)	1×	482 mL
L-ascorbic acid-2-phosphate (64 mg/mL)	64 μg/mL	500 μL
Insulin-Transferrin-Selenium (ITS-G, 100×)	2×	10 mL
HEPES (1 M)	15 mM	7.5 mL
**Total**	**N/A**	**500 mL**

Can be stored at 4°C for up to 2 weeks.

To prepare L-ascorbic acid-2-phosphate stock solution (64 mg/mL), resuspend 500 mg in 7.81 mL sterile MilliQ water, sterile filter, aliquot and store at −20°C for up to one year.

**Table:** E6-NI gradient medium-1

Reagent	Final concentration	Amount
E6 (1×)	0.66×	8 mL
NM (1×)	0.33×	4 mL
SB431542 (10 mM)	10 μM	12 μL
LDN-193189 (2.5 mM)	0.25 μM	1.2 μL
**Total**	**N/A**	**12 mL**

Can be stored at 4°C for up to 5 days.

**Table:** E6-NI gradient medium-2

Reagent	Final concentration	Amount
E6 (1×)	0.33×	4 mL
NM (1×)	0.66×	8 mL
SB431542 (10 mM)	10 μM	12 μL
LDN-193189 (2.5 mM)	0.25 μM	1.2 μL
**Total**	**N/A**	**12 mL**

Can be stored at 4°C for up to 5 days.

## Step-by-step method details

### Preparation of iPSCs and neural induction

**Timing: 7 days**

**Figure 1:**
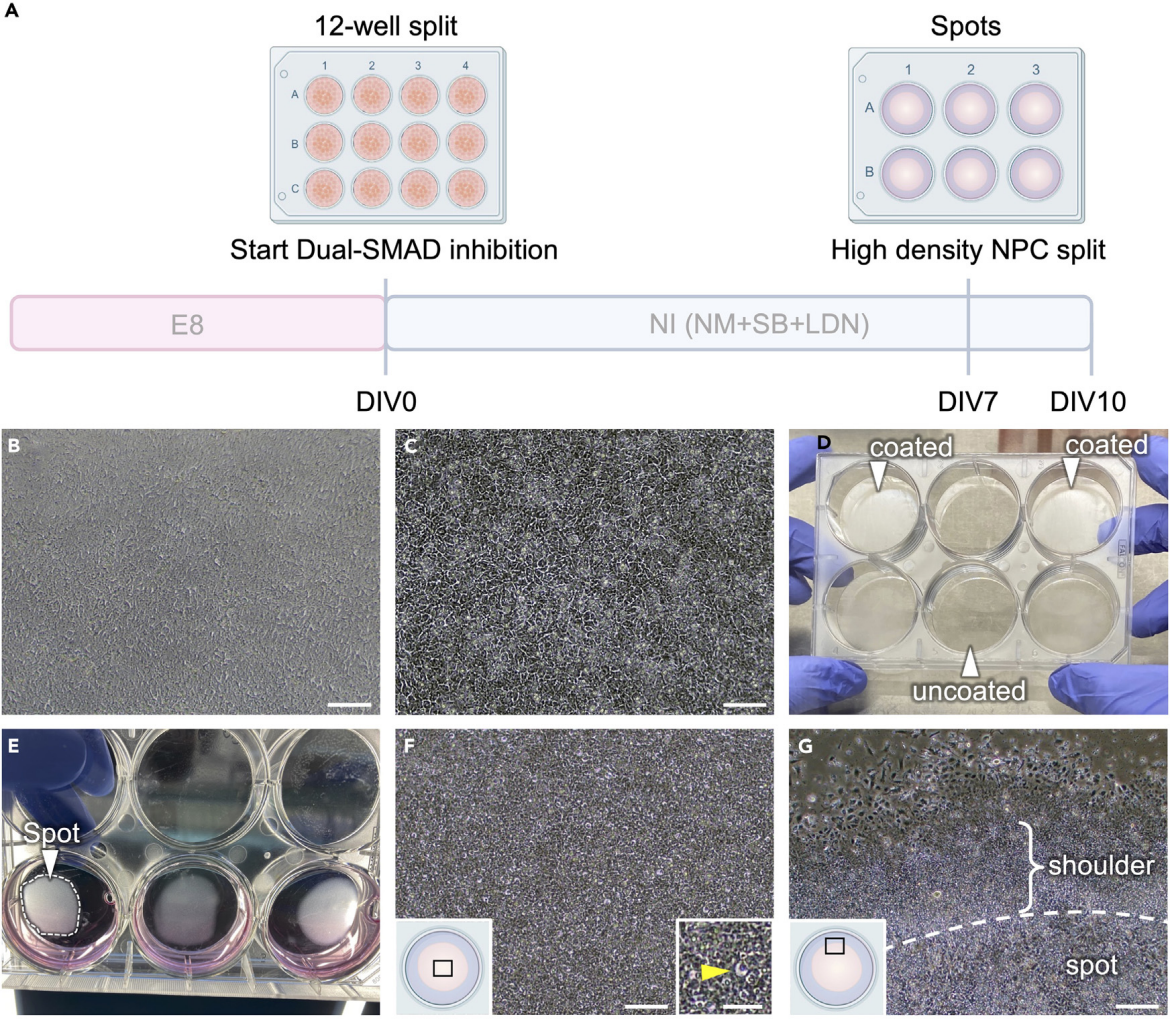
Plating cells in high-density spots to induce neural rosette formation (A) Timeline for the first part of the differentiation up to day-in-vitro (DIV) 10. Human iPSCs kept in E8 Flex media are split into 12-well plates on DIV0, and neural differentiation is induced by dual-SMAD inhibition with SB431542 and LDN193189. On DIV7, early neural progenitor cells (NPCs) are split into high-density spots in 6-well plates to continue high-density differentiation and promote neural rosette formation. (B) Cells in 12-well plate on DIV1 of neuronal differentiation. Note the confluent and uniform distribution and appearance of the cells in a monolayer. Scale bar 100 μm. (C) Cells in 12-well plate on DIV7 of neuronal differentiation, just before the split into spots. Note the high density and uniform appearance of the cells without much cell death or formation of clumps. Scale bar 100 μm. (D) Dried POL plate just before making spots. Note the even crystalline (white) sheet in coated wells on the sides vs. uncoated wells in the middle (arrowheads). (E) Spots on 6-well plate the day after plating (arrowhead/dashed line). Note the uniform, dense appearance without holes. (F) NPCs in the center of the spot 3 days after plating (for location see illustration). Distribution of cells is still very even, and no clear rosettes are visible yet. Note the bright, half-moon-shaped structures of cells indicating correct differentiation (insert in the bottom right, yellow arrowhead). Scale bar 100 μm, insert 50 μm. (G) Edge of the spot around DIV10 (for location see illustration). Cells at the spot edge begin forming a “shoulder” of densely packed cells, separating cells inside the spot from outside of the spot. Cells on the inside will later form rosettes, while the shoulder and cells outside may differentiate aberrantly and should not be used in the next steps of the protocol. Scale bar 100 μm.

In this first step, neural induction from iPSCs is initiated using dual-SMAD inhibition based on previously published studies.^6^^,^^7^^,^^8^ A schematic overview of this step is included in Figure 1A.

1. Prepare required number of iPSCs on vitronectin-coated 6-well plates as described above.
2. Prepare required number of GTX-coated wells of a 12-well plate; as a starting point coat two to three wells of a 12-well plate per well of a 6-well plate of iPSCs.

**CRITICAL:** Clean off spontaneously differentiated cells in the iPSC culture thoroughly prior to starting the neural induction, as these will inhibit neuronal differentiation. Aberrant differentiation of iPSCs should be <5%. Troubleshooting 1.

3. Split iPSCs into wells of a GTX-coated 12-well plate when they are 70%–80% confluent; this day is defined as Day-in-vitro 0 (DIV0).
  a. Add 0.5 mL Accutase to the well with iPSCs.
  b. Incubate 5–8 min at 37°C until most cells detach when shaking/tapping the plate; if unsure, monitor under a microscope that cells are detaching.
  c. Add 1 mL DMEM-F12, gently triturate to single cells with a P1000 pipette and count cells.
  d. Transfer required amount of cell suspension (1 million cells per well of a 12-well plate) into a 15 mL conical tube and spin 4 min at 170 *g*.
    ***Note:*** All centrifugation steps are performed with these parameters at RT unless indicated otherwise.
  e. Aspirate media and resuspend pellet to a single cell solution at 1 million cells/mL in NI supplemented with RI (10 μM).
  f. Plate 1 mL of cell suspension (1 million cells) per well of a 12-well plate on the prepared, GTX-coated plates (resulting in a cell density of 260,000 cells/cm^2^).
  g. Evenly distribute cells by moving the plate back and forth in two directions.
    ***Note:*** Cells should have formed a confluent monolayer on the next day (Figure 1B). Increase plating density the next time if cells are not confluent on this day and decrease density if issues with detaching cells occur later.
    ***Note:*** If required, cell clumps can be carefully removed using a vacuum pump, but plating density and resuspension to single cells should be optimized to avoid formation of clumps, also see troubleshooting 2.

4. Change NI media every day until DIV7.
  a. The first days, feed 1 mL NI per well of a 12-well plate.
  b. Increase media volume to 1.5–2 mL on DIV5-7 to ensure sufficient supply with growth factors and nutrients.
    ***Note:*** Sufficient nutrient supply will improve the quality of the high-density spots in the next step.
    ***Optional:*** use E6 – NI gradient for difficult-to-differentiate cell lines on DIV0 and DIV1 starting with the split; also see troubleshooting 3 for more information
    i. On DIV0, perform split as described above using 1 mL E6-NI gradient medium-1 instead of NI.
    ii. On DIV1, change medium to 1 mL of E6-NI gradient medium-2.
    iii. On DIV2, change medium to NI and continue with the standard protocol.
      ***Note:*** Depending on the cell density and iPSC line, each well of a 6-well plate of iPSCs will yield 2–4 million cells. We recommend optimizing this first split (starting cell density, number of cells per well of a 12-well plate) for each cell line depending on its proliferative capacity.

### Neural rosette formation in spots

**Timing:** around 1 week

In this step, neural precursor cells (NPCs) are plated in spots to achieve maximum cell density while allowing sufficient nutrient supply when fed once per day. High density plating is crucial to achieve high quality cortical neuron differentiations. A schematic overview of this step is included in Figure 1A.

5. On DIV6 (=one day before the split), coat wells of a 6-well plate with 1 mL of each PO and L as described under “Materials and Equipment”. Depending on the proliferative capacity of the iPSC line, one full 12-well plate is usually split into 4–8 wells of a 6-well plate.
6. On the day of the split, the cells should look as depicted in Figure 1C.
7. On DIV7 (=day of the split), aspirate coating solution completely and leave the plate open in the laminar flow hood to dry for about 20–30 min, turn around once after 10–15 min to allow even drying.

***Note:*** A crystalline sheet will appear, there should be no clear gaps in the sheet (Figure 1D).

8. Perform the high density split into spots on DIV7, also see Methods video S1.
  a. Aspirate the medium and add 0.5 mL Accutase to the cells, incubate 10 min at 37°C.
  b. Add 1 mL DMEM-F12 (10 μM RI can be added to DMEM-F12 when more than one plate needs to be split simultaneously).
  c. Wash off cells with a P1000 pipette to obtain a single cell suspension, pool the suspension in a 15 mL conical tube and count the cells.
  d. Spin down cells, aspirate supernatant, resuspend cells in appropriate amount of NI/RI to get 30 million cells per mL, make sure to obtain a single cell suspension.
  e. Put one 250–350 μL drop per well on dried POL 6-well plates. The cell suspension should stay in the middle of the plate and not disperse (Methods video S1), do not introduce air bubbles.
  f. If necessary, spread the spot slightly by gently tilting and tapping the plate, such that its diameter is about 1/3 to 1/2 of the well diameter (depending on the spot volume).
    ***Note:*** Avoid contact between the cell solution and the edges of the well, as this would pull cell solution towards the edge, resulting in deformed spots with lower cell density and uniformity.
  g. Let cells attach for 60 min at RT without adding additional media; put the lid on the plate and keep inside the hood (i.e., do not put the cells into the incubator) to avoid dislodging the spot while cells are attaching.
  h. After 60 min, add 2 mL NI/RI very gently to the wall of the plate with a serological pipette, tilt the plate very gently to spread out the NI, ensure that cells stay in the spot and are not washed off (Figure 1E).
9. The next day, shake or tap the plate to dislodge any non-attached/dead cells and replace the medium with fresh 2 mL NI; the spots should stay on the plate and form a dense, uniform cell layer (Figures 1E and 1F).
10. Change NI media every day, 3–4 mL feeds are sufficient to bridge one weekend day.
11. On DIV10, switch to 2 mL NM media.
12. Starting from DIV10, for subsequent feeds change NM media every day, 3–4 mL feeds are sufficient to bridge one weekend day.
  a. Around DIV10, a “shoulder” of partially non-cortical cells forms at the very edge of the spots (containing neural crest cells and other cells) while the high-density cells in the middle of the spots soon start forming neural rosettes (Figure 1G).

**CRITICAL:** The quality of cells in the 12-well plate before making spots needs to be very high. The cells should be in a uniform layer and should not peel off the well or start dying (see Figures 1B and 1C) – if they do, the starting cell density in the 12-well plates needs to be optimized. For more proliferative iPSC lines, the day of making the spots can be shifted to DIV6 to ensure cells are confluent in the 12-well plate after plating but do not peel off on the day of making the spots. Troubleshooting 4

**CRITICAL:** Drying of the POL-coated plates is critical for the successful generation of spots. It is crucial to have an even crystalline sheet appear on the bottom of the plates, as spots will peel off if they are placed onto gaps in the sheet. The relative hydrophobicity of the sheet will contain the area covered by cells which allows for the high cell densities required for successful neuron differentiation. Troubleshooting 4

**Figure 2:**
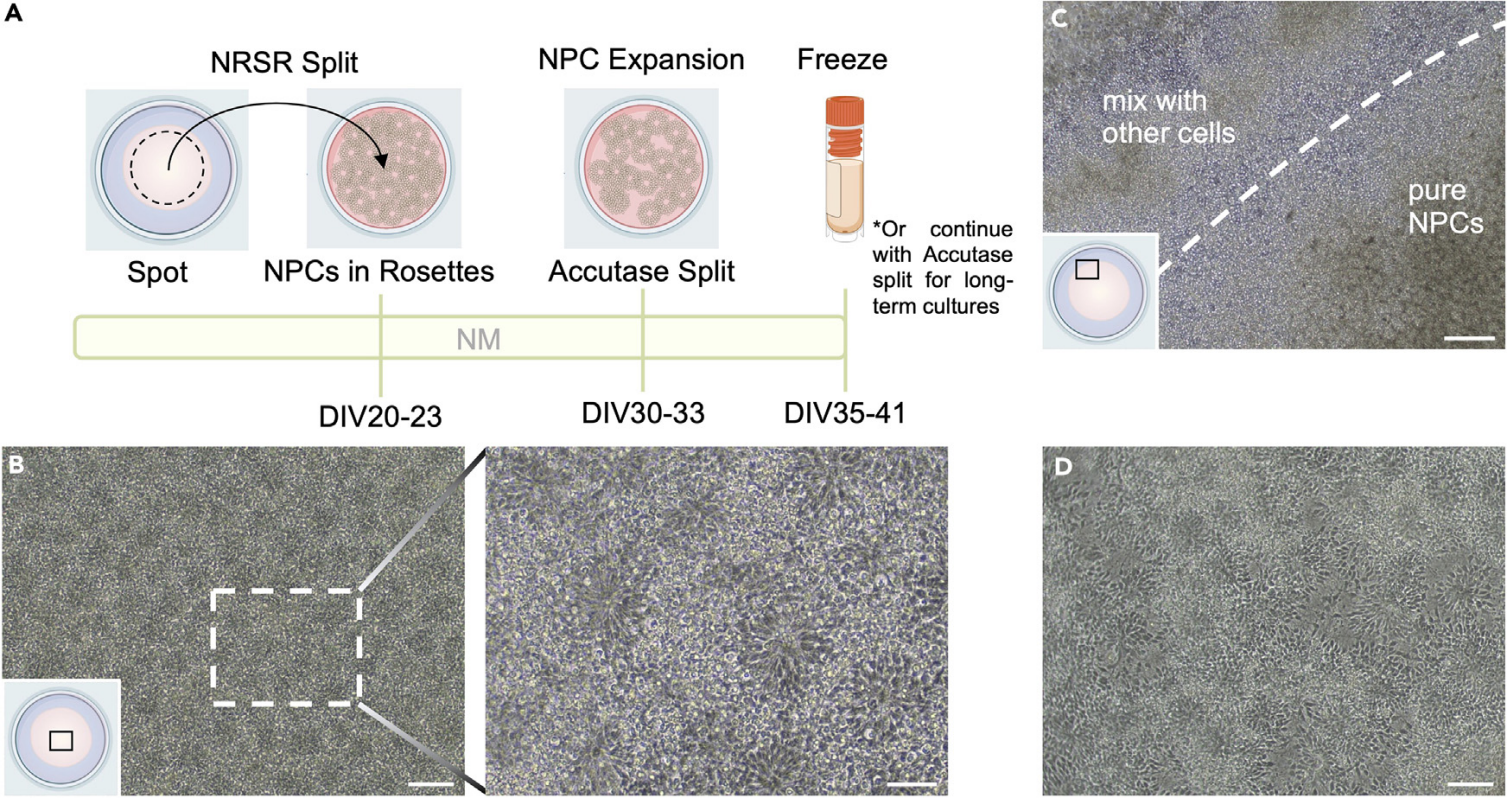
Expansion and enrichment of neural rosettes (A) Timeline for the second part of the differentiation. Early NPCs in spots start forming rosettes, which are collected around DIV23 using neural rosette selection reagent (NRSR) and manual selection. Rosettes are further expanded by another split using Accutase around DIV30. Late NPCs and early neurons are then either frozen for cell banking around DIV37 or split again with Accutase into single cells for subsequent long-term culture. (B) Rosette formation in spots on DIV18. **Left:** Overview image (for location in the spot see illustration). Note uniform, chess-board-like structure of rosettes. Scale bar 100 μm. **Right:** Magnification of indicated area showing typical structure of single rosettes. Scale bar 50 μm. (C) Accumulation of rosettes around DIV23. Shown is the edge of the spot (see location in the illustration). Note multiple “circles” and brown appearance of densely packed, accumulated rosettes inside the spot (bottom right). At this stage, spots commonly have a clearly defined shoulder (dashed line), which separates the desired, pure NPCs in rosettes from neural crest and other aberrantly differentiated cells. The shoulder will be used as guidance to manually isolate the rosettes during the NRSR split along the dashed line (see Methods video S2). Scale bar 250 μm. (D) Isolated rosettes around DIV28 (after NRSR split). Only rosettes and migrating NPCs are visible (dark, compact and rounded cells), note outgrowth of neurites from late NPCs/early neurons. Scale bar 100 μm.

***Optional:*** If not enough cells are available to prepare spots at the indicated density (30 million cells/mL) and volume (250–350 μL), spots can be made with smaller volumes but same density. This will however disproportionally decrease final rosette yield, as the area of the spot "shoulder" - containing potentially aberrantly differentiated cells - will be increased relative to the area of rosettes on the inside which are used for further differentiation (see Figure 2B and step 15 below).

***Optional:*** If cell density is low on DIV10 (e.g., holes are forming) when changing to NM, bFGF (20 ng/mL) can be added to NM for the next 2 feeds. Troubleshooting 5

***Note:*** On DIV13-15, the rosettes in the spots can be used to differentiate astrocytes using the serum-free protocol by Perriot et al.^9^

Methods video S1. Generation of high-density spots on dried, POL-coated plates on DIV7 of the protocol, step 8

### Expansion of neural rosettes

**Timing:** around 3 weeks

In this step, the population of NPCs is expanded to increase the number of resulting neurons at the end of the differentiation protocol. To achieve this, it is crucial to maintain the integrity of neural rosettes. A schematic overview of this step is included in Figure 2A.

13. When neural rosettes appear, usually around DIV15 (Figure 2B), feed cells for 2 consecutive days with 2 mL NM supplemented with bFGF (20 ng/mL) (= NM + bFGF), to support proliferation and expansion of the rosettes.
14. For subsequent feeds, change NM media every day, 3–4 mL feeds are sufficient to bridge one weekend day.
15. When rosettes start accumulating (usually around DIV20–23: the culture develops brownish, dense areas/circles visible under the microscope, Figure 2C), split cells with NRSR (STEMdiff Neural Rosette Selection Reagent), also illustrated in Methods video S2.
  ***Note:*** NRSR is a reagent that is designed to specifically dislodge rosettes without breaking them apart, resulting in separation of rosettes from undesired cells. Here, it is used in combination with manual selection of neural rosettes inside the spot to increase the purity of the culture and enrich for NPCs; using this procedure, only one NRSR split is required to obtain pure neuron cultures.
  a. Depending on the density and size of the spots, split the spots in a 3:2 to 3:4 ratio onto POL-coated 6-well plates; as a starting point split one spot into one well of a 6-well plate.
  b. Aspirate media.
  c. Add 1 mL NRSR, incubate 30–60 min at 37°C (optimize time for cell line used).
  d. Aspirate NRSR and add 1.5 mL NM.
  e. Manually isolate rosettes from inside the spot using a pipette tip (Methods video S2 and Figure 2C), do not touch the “shoulder” of the spot or cells outside the spot.
  f. Collect dislodged rosettes in a 15 mL conical tube, triturate slightly to break up larger clumps (5–10× with Pasteur pipette or P1000 if more careful), make sure to not dissociate the neural rosettes into single cells.
  g. Spin down cells, remove supernatant, carefully resuspend cells in NM + bFGF, triturate 1–2× with 5 mL serological pipette.
  h. Plate 2 mL of the cell suspension per well of a POL-coated 6-well plate.
    ***Note:*** The goal of this step is to keep rosettes intact but break up aggregates of rosettes and give them more space for growth (Figure 2D). Rosettes need to be grown at high density without generating too many single cells, as these will fall out of cell cycle, reducing final cell yield. Therefore, special attention should be paid when triturating the cell suspension. Troubleshooting 6

16. Feed with NM + bFGF on the next day.
17. For subsequent feeds, change NM media (without bFGF) every day to every other day.
18. Cultures should contain single rosettes and small aggregates of rosettes, but few to no other cell types or large clumps of cells, the NPCs (radial-glia-like cells) in the middle of the rosettes will keep dividing and the cultures will become denser (Figure 2D).
19. When rosettes become almost confluent (usually around DIV30–33), split again to give cells more space, also see Methods video S3.
  a. Split 3 wells into 3–5 wells, depending on density and proliferative capacity of the cell line.
  b. Incubate with 0.5 mL Accutase for 4 min at 37°C, do not aspirate, add 1 mL NM.
  c. Wash cells off with plastic Pasteur pipette or P1000 pipette.
  d. Pool and triturate gently with Pasteur pipette or P1000 to separate rosettes from each other; avoid making single cells.
  e. Spin cells down, resuspend in fresh NM + bFGF and plate in 2 mL per well of a 6-well plate.
20. Feed with NM + bFGF on the next day.
21. For subsequent feeds, feed with NM media every day to every other day.

***Note:*** The cultures should look like the rosettes before the Accutase split, but less dense with more neurites (Figure 2D). Troubleshooting 6

Methods video S2. Manual selection of highly-enriched NPCs using NRSR, step 15

Methods video S3. Accutase Split of NPCs, steps 19 and 28

### Freezing and thawing of neural rosettes

**Timing: 15 min for each freezing or thawing procedure, 1 day to coat plates before thawing**

In this step, we describe how to freeze the neural rosettes for long-term storage in a liquid nitrogen tank, as mature cortical neurons cannot be frozen. We also describe how to thaw the neural rosettes. A schematic overview of this step is included in Figure 2A.

**Pause point:** Rosettes can be frozen at this point and stored in liquid nitrogen for extended periods of time (months to years). Long-term culture of the differentiated neurons can be resumed at any later timepoint, allowing for synchronization of different cell lines and cell types for long-term culture.

22. Freezing of neural rosettes works best between DIV35 and DIV41.
  a. Add 0.5 mL Accutase to the cells, incubate for 4 min at 37°C.
  b. Do not aspirate Accutase, add 1 mL NM and triturate cells gently with Pasteur pipette without breaking up rosettes, collect cells in a 15 mL conical tube.
  c. Spin down cells and resuspend the NPCs in 1 mL cold Freeze Medium for Neural Rosettes for every 1–2 collected wells, depending on cell density; triturate 2–3× with 5 mL pipette.
  d. Transfer cell suspension to cryovials and freeze vials with 1 mL cell suspension in prechilled Mr. Frosty at –80°C, save a drop to genotype cells if needed.
  e. The next day, transfer frozen rosettes to liquid nitrogen tank (−80°C is not sufficient for long-term storage).

***Optional:*** To facilitate planning, count cells before freezing by triturating a small, representative aliquot to single cells. Freeze down NPCs at about 10 million cells per cryovial to thaw them into one well of a 6-well plate. Document yield (i.e., counting the neurons obtained from the differentiation) and/or quality of the cells (e.g., by taking representative brightfield images) to optimize future differentiations and adjust subsequent experimental setups.

23. Thawing of neural rosettes.
  a. Coat required number of wells/plates with POL the day before thawing.
  b. Prepare 9 mL Neurobasal media in 15 mL conical tubes, one tube per vial thawed.
  c. Prepare Thawing Medium for Neural Rosettes, use 2 mL per vial thawed (if plating in wells of a 6-well plate).
  d. Quickly thaw frozen rosettes in a 37°C water bath until only a small clump of ice remains.
  e. For each vial, slowly add 0.5 mL of the prepared Neurobasal media into the cryovial to dilute Freeze medium, carefully pipette the rosettes up and down 1–2× with Pasteur pipette (if no large clumps are present) or the P1000 pipette (if large clumps are present).
  f. Gently transfer the thawed cell suspension to the prepared tubes with Neurobasal media.
  g. Spin down cells and aspirate supernatant.
  h. Resuspend cells in 2 mL Thawing Medium for Neural Rosettes using Pasteur pipette (P1000 if there are larger clumps) and plate into one well of a 6-well plate (assuming cells were frozen at ∼10 million cells/vial), shake plate to distribute cells and transfer to incubator.
24. On day post thaw (DPT) 1, if the media is yellow, add 1–2 mL fresh NM + bFGF on top.
25. On DPT 2, do a full feed with 2 mL NM + bFGF.
26. Feed 2 mL NM on following days as needed.
27. On DPT 4–7, split the neurons for long-term culture as described below.

***Note:*** Neuronal long-term cultures can also be generated directly from differentiated cells without freezing. In order to do so, perform another Accutase split as described in points 19–21 between DIV37-DIV41, and mature the rosettes further until DIV 43–48 when plating of the neurons for long-term cultures is performed (see below).

***Note:*** as alternative to RVC, both RI or CEPT^10^ can be added to thawing medium.

***Optional:*** To ensure cortical identity of the differentiated neurons, stainings for neuronal markers can be performed as we describe in the Expected Outcomes section. If other neuronal cell types are detected, refer to troubleshooting 7.

### Long-term culture of differentiated cortical neurons

**Timing: 1 day to coat plates, 20–45 min for splitting the cells, 1 month to 1 year for long-term culture**

In this step, we describe how to plate the neurons in different well formats to ensure stable culture of the cells for up to 12 months.

28. Split rosettes on DPT 4–7 (or alternatively 4–7 days after the last split) onto 6/12/24-well, POL-coated plates; this is usually DIV41–47.
  ***Note:*** Cells should have recovered from thawing by rearranging into rosettes, with early neurons being generated as seen by neurite formation. The space between rosettes should be free of other cell types and dead cells that may be visible after thawing should be gone after media changes.
  a. Plate the cells at these recommended cell densities (160,000–210,000 cells/cm^2^) to obtain uniform neuronal networks:
    i. 24-well plate: 300–400 k cells/well.
    ii. 12-well plate: 0.6–0.8 mio cells/well.
    iii. 6-well plate: 1.5–2.0 mio cells/well.
      ***Note:*** Neuronal survival after the final split may vary. We recommend aiming for the highest possible density in the provided range that still allows uniform neuron growth without major clumping. If the density is too high, clumping will occur due to clustering of the neurons.
  b. Prepare required number of POL-coated wells/plates as described above.
  c. Prepare the following before the split:
    i. one 15 mL tube with 4 mL NM per well of a 6-well plate with rosettes.
    ii. required amounts of Accutase (0.5 mL per well of a 6-well plate with rosettes).
    iii. the required amounts of plating medium: NB+/B27+ supplemented with DAPT (10 μM) and RVC (1:100); for plating: use 2 mL/ 1 mL / 0.5 mL for wells of 6-well/12-well/24-well plates respectively.
  d. Aspirate NM, add 0.5 mL Accutase to rosettes and incubate 6–10 min at 37°C (depending on density and morphology).
    ***Note:*** If unsure about the incubation time, check under the microscope that neurons are detached, and neurites are retracted to not harm the cells when triturating; if this is not the case increase the incubation time for Accutase.
  e. Add 1 mL NM to Accutase with a P1000 pipette, thereby detaching the cells.
  f. Carefully triturate 8–12× with P1000 to break up any remaining rosettes and generate a single cell suspension; making single cells works best by holding the plate at a 45°- angle and pipetting cells in and out of the P1000 tip while moving the tip left and right (Methods video S3).
    **CRITICAL:** When triturating, stay inside the cell suspension, avoid generating air bubbles and focus on large clumps in the suspension. Do not overtriturate cells, if small clumps remain after ∼12× triturating, use the optional straining step below to remove them.
  g. Collect the cells in the prepared 15 mL tube with 4 mL NM (cells are diluted to facilitate counting and reduce harsh contact of the neurons with the plastic).
    ***Optional:*** Transfer single cell suspension through a 70 μm cell strainer into the prepared 15 mL tube to remove any remaining large clumps from the suspension. Large clumps should not remain in the suspension as they often contain proliferative NPCs and thus impair stable long-term neuronal culture. Pre-wet the cell strainer and the wall of the 15 mL tube with media to minimize cell loss and death.
  h. Count cells (one well can contain 5–20 million cells depending on cell density).
  i. Transfer the required number of cells into a new tube, spin down.
  j. Aspirate supernatant, resuspend and gently triturate with P1000 pipette in 1 mL Plating medium until no clumps are visible anymore (usually 4–6×).
  k. Add required media volume to reach recommended cell density and plate quickly (occasionally mix the tube when plating many wells).
    ***Note:*** DAPT inhibits Notch signaling and thereby promotes terminal differentiation of NPCs into neurons.
    ***Note:*** If neurons are to be plated onto glass coverslips for imaging, the coverslips need to be pre-treated with 30% HCl at least 12 h to roughen the glass surface. Afterwards, wash coverslips 4–5× with de-ionized water to fully remove the acid. Store coverslips in 100% Ethanol. Dry off Ethanol and coat the coverslips with POL as described above before use.
    ***Note:*** Cells are very sensitive at this stage, therefore it is important to work as quickly as possible, minimize trituration, and to not introduce air bubbles. Always add cells directly into media if possible.

29. Extended culture of neurons over longer time periods (1 month–1 year).
  a. Feed the cells 2 days after plating with NB+/B27+ with DAPT.
    i. Before the feed, tap the plate to dislodge debris and dead cells into the supernatant.
  b. On day 3, do a half-feed.
    i. Prepare the required amount of NB+/B27+ medium and supplement it with 10 μM DAPT and 5 μM 5-Fluorouracil and 5 μM Uridine (pre-mixed as 5FU) as indicated in the “Materials and Equipment” section.
      ***Note:*** 5-Fluorouracil is added to kill residual dividing cells, while Uridine is added to minimize the effect of 5-Fluorouracil on transcription and thus reduce toxicity in non-dividing cells.
  c. Consecutively do half feeds with NB+/B27+ supplemented with DAPT and 5FU two to three times per week.
    i. DAPT should be added for a total of 7 days (starting with the day of plating).
    ii. 5FU should be added for a total of 10–14 days (starting at the feed 3 days after plating).
      ***Note:*** If a lot of cell death occurs during 5FU treatment, tap the plate to dislodge debris and do a full feed.
      ***Note:*** Ensure that media volumes stay constant during half feeds by briefly tilting plates after removing media and checking contents in individual wells, media in peripheral wells may evaporate faster.
  d. After the 5FU treatment ends, switch to half feeds with NB+/B27+ two to three times per week.
  e. Cells will start growing neurites the day after plating; after two weeks, the cells will have formed a dense neuritic network; there should be over 95% neurons and no large patches of other cell types, some astrocytes may be present 1–2 months after plating.
    ***Note:*** We tested long-term neuron culture with different neuronal media. Neurons can also be fed with NB/B27, NB/B27 PLUS and will survive for long-term, but in our experience, the cultures are more stable in NB PLUS/B27 PLUS in terms of survival for extended culture periods of >4 months. We did not test Brainphys for long-term culture, because we noted increased contamination with astrocytes.

## Expected outcomes

**Figure 3:**
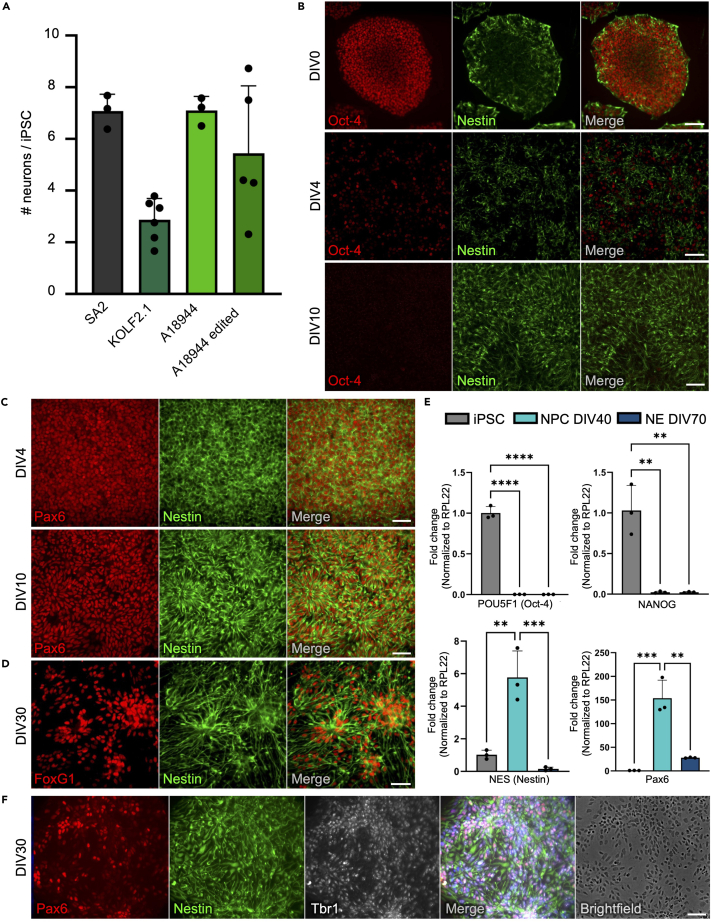
Quality controls confirming high yield and successful neural induction (A) Differentiation yields of different iPSC lines (shown as resulting late NPCs/early neurons on DIV40 per iPSC used to start the differentiation). SA2/KOLF2.1/A18944: WT cell lines, A18944 edited: Cell line gene-edited using CRISPR/Cas9. Data represented as Mean ± SD, n = 3–6 (each n represents an independent differentiation). (B) Immunofluorescence (IF) stainings of iPSC and NPCs on day-in-vitro (DIV) 0 (top), 4 (middle) and 10 (bottom) demonstrating gradual loss of pluripotency marker Oct-4 and increased expression of proliferation-associated Nestin. Scale bar 100 μm (DIV0 and DIV4) and 30 μm (DIV10). (C) IF stainings of early NPCs on DIV4 (top) and 10 (bottom) showing expression of NPC markers Pax6 and Nestin. Scale bar 30 μm. (D) IF staining on DIV30 confirming expression of forebrain marker FoxG1 in Nestin-positive NPCs. Scale bar 30 μm. (E) RT-q-PCR analysis on iPSCs (DIV0), late NPCs/early neurons (DIV40) and neurons (NE, DIV70) showing downregulation of pluripotency factors Oct4 and Nanog from DIV0 to DIV40/70. NPC markers Nestin and Pax6 are upregulated over the course of differentiation from iPSCs (DIV0) to NPCs (DIV40) and downregulated during neuronal maturation from DIV40 to DIV70. Data are represented as Mean ± SD. ∗∗p < 0.01, ∗∗∗p < 0.001, ∗∗∗∗p < 0.0001, one-way ANOVA., n = 3 (each n represents an independent differentiation). (F) IF staining of late NPCs/early neurons on DIV30 confirming expression of NPC markers Pax6 and Nestin as well as deep-layer neuron marker Tbr1. Scale bar 30 μm.

The presented protocol yields highly pure cultures of cortical forebrain neurons by ensuring constantly optimal differentiation conditions, e.g., uniformly high cell density, enrichment of neuronal rosettes, and removal of proliferative precursor cells. We tested our protocol in several different iPSC lines both before and after CRISPR/Cas9-mediated genomic edits. We can differentiate all tested iPSC lines robustly into cortical neurons, as shown in Figure 3A.

To ensure high quality of the differentiations and to confirm neuronal fate, we recommend performing quality control experiments, especially when establishing the differentiation of a new iPSC line. For this purpose, we describe the expected outcomes of the differentiation and include typical markers that are expressed during the differentiation. Additional quality control parameters that can be used to assess the generated neurons can be found in previously published studies.^6^^,^^7^^,^^8^

**Figure 4:**
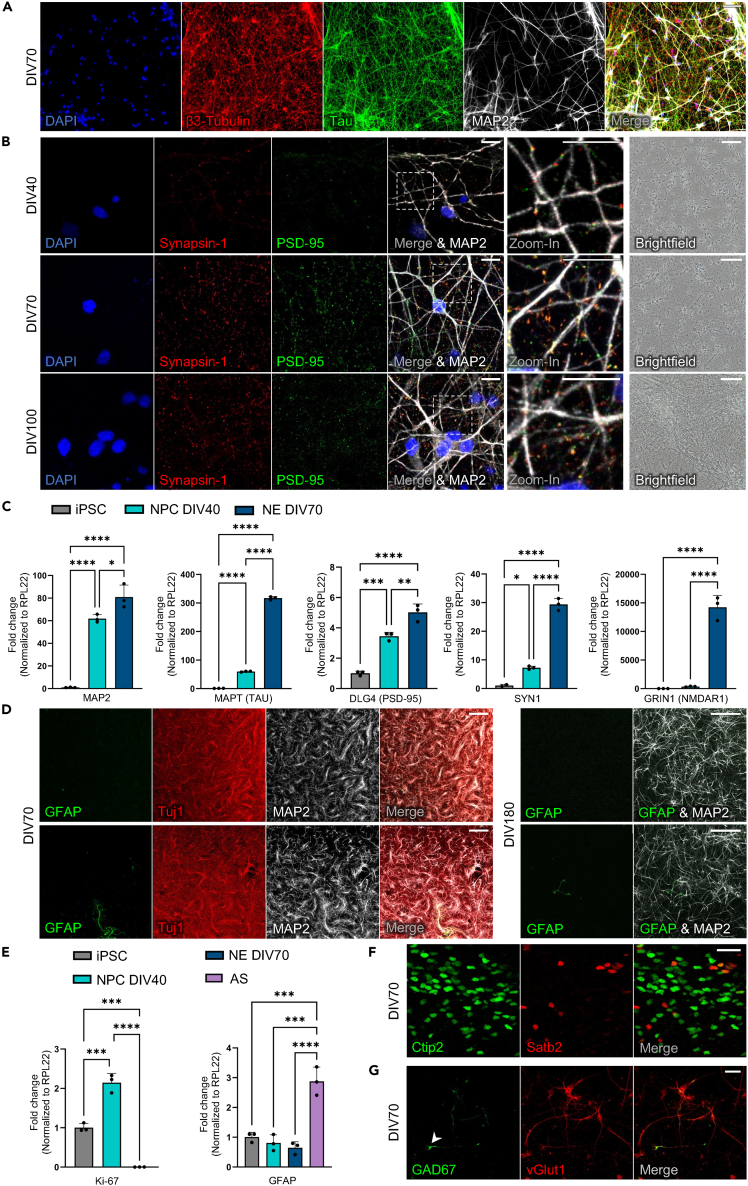
Characterization of differentiated neurons confirming maturation, high purity, and cortical identity (A) IF staining of cortical neurons on DIV70 confirming expression of neuron markers β3-Tubulin, Tau, and MAP2. Scale bar 50 μm. (B) IF staining on DIV 40 (top), 70 (middle) and 100 (bottom) showing gradual formation of synapses as seen by colocalization of presynaptic Synapsin-1 and postsynaptic PSD-95. Dendrites are stained by MAP2. Zoomed-in regions are marked by dashed square in merged image. Scale bar 10 μm. Brightfield images on the right showing the increasing density of the neuritic network in the cultures over time. Scale bar 100 μm. (C) RT-q-PCR analysis on iPSCs (DIV0), late NPCs/early neurons (DIV40) and cortical neurons (DIV70) showing upregulation of neuronal markers MAP2, Tau, PSD95, Synapsin-1 and NMDAR1 over time. Data are represented as Mean ± SD. ∗p < 0.05, ∗∗p < 0.01, ∗∗∗p < 0.001, ∗∗∗∗p < 0.0001, one-way ANOVA, n = 3 (each n represents an independent differentiation). (D) IF staining of cortical neurons (Left: DIV70, Right: DIV180) confirming minimal contamination of cultures with astrocytes. **Left:** Note the only astrocyte present on entire 12 mm coverslip in bottom image. **Right:** In total 7 astrocytes were found on entire 12 mm coverslip. Scale bars 500 μm. (E) RT-q-PCR analysis as in C) showing absence of Ki67 expression in neuron cultures at DIV70, indicating a postmitotic culture without proliferating cells; and low levels of GFAP expression at similar levels to iPSCs, corroborating high purity and minimal contamination with astrocytes. A pure astrocyte (AS) culture (purple) was used as positive control. Data are represented as Mean ± SD. ∗∗p < 0.01, ∗∗∗p < 0.001, ∗∗∗∗p < 0.0001, one-way ANOVA, n = 3 (each n represents an independent differentiation). (F) IF staining showing expression of deep-cortical-layer marker Ctip2 and mid-cortical-layer marker Satb2 on DIV70. Scale bar 30 μm. (G) IF staining of neurons on DIV70 showing that most cells are vGLUT1-positive, glutamatergic neurons; a small percentage of GAD67-positive, GABAergic neurons is also present. Note single GAD67-positive neuron in field of view (arrowhead). Scale bar 30 μm.

In the first days of neuronal differentiation, the cells lose pluripotency markers such as Oct4 and NANOG and increase expression of neuroepithelial and cortical NPC markers such as Pax6, Nestin, and FoxG1 (Figures 3B–3E). The NPCs proliferate and form neural rosettes by day 12–15, which are then purified and expanded. After the final split (around DIV41–47), the separated, single cells lose expression of neural progenitor markers such as PAX6 and Nestin and upregulate neuronal markers such as MAPT, MAP2, and NMDAR1 (Figures 3E, 4A–4C). Overall, the combination of weekly splits as well as DAPT and 5FU treatment leads to a highly homogeneous population of neurons, by removing already postmitotic cells with extended processes during splits, and those still dividing after the final split by mitotic inhibition, respectively. Thus, terminal differentiation occurs within a narrow time frame, finally yielding a highly pure culture of cortical forebrain neurons.

Neurons differentiated from iPSCs using this protocol express typical markers such as MAP2, β3-Tubulin, and Tau directly after neuron differentiation both on mRNA and protein level (Figures 4A and 4C). Synapses are formed from the beginning and increase over time while the cultures mature further (Figures 4B and 4C). During this maturation, extensive neuritic networks are formed, neuronal markers are further upregulated, and post-mitotic cortical neurons dominate the culture while contaminating astrocytes are minimal (Figures 4C–4E).

Mimicking layer formation in the human cortex, the protocol yields lower-layer, CTIP2-positive neurons and mid-layer, SATB2-positive neurons (Figure 4F), with a larger number of deep-layer neurons. This is because deep layer neurons are formed first in human development with mid- and upper-layer neurons following afterward, which we block by DAPT and 5FU treatment. Performing these treatments earlier^11^ or later^7^ will alter the fraction of neurons from different layers. Cortical neurons are largely glutamatergic (vGLUT1-positive), with a small proportion of GAD67-positive GABAergic cells present (Figure 4G).

**Figure 5:**
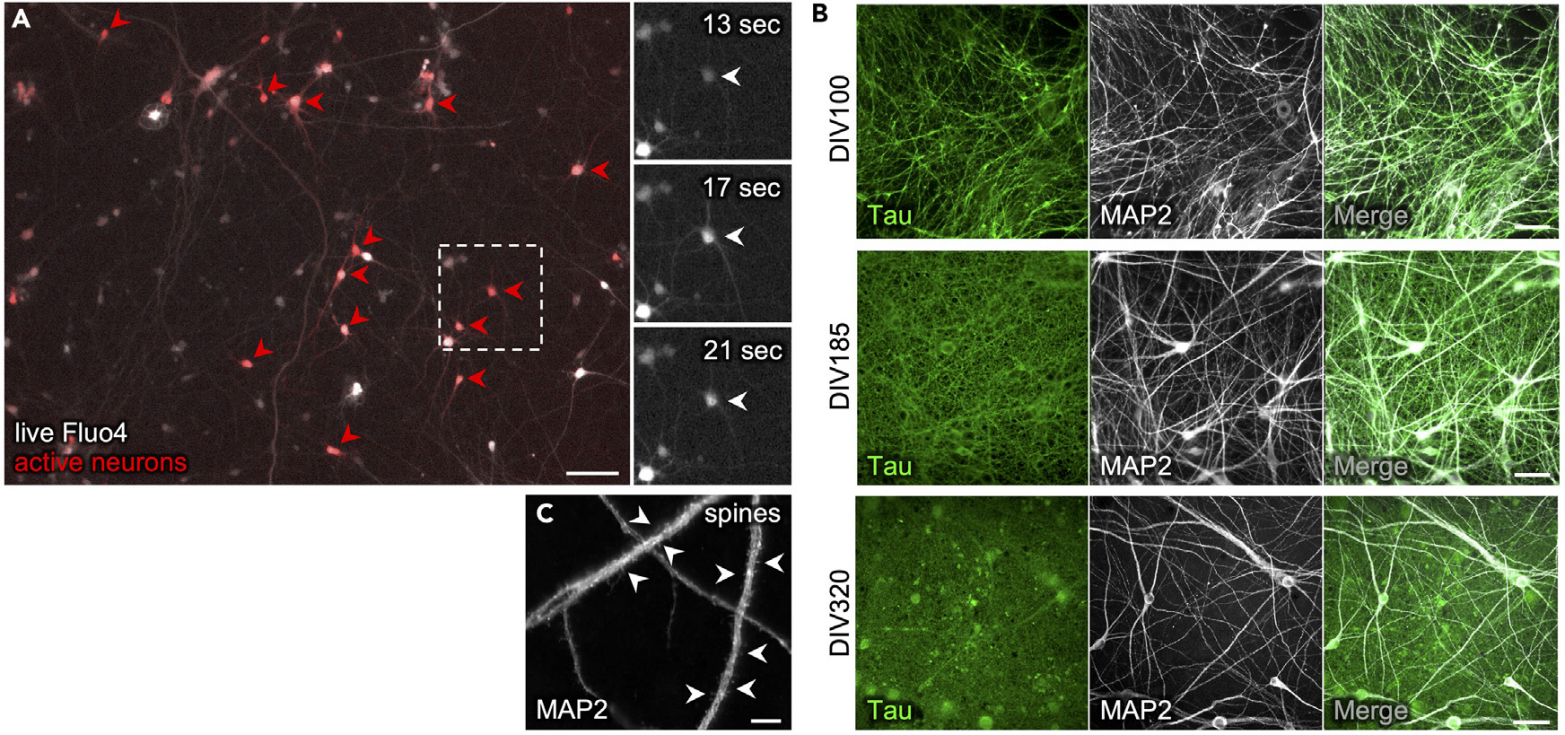
Long-term culture of differentiated neurons shows increasing network density and neuronal activity (A) Still image from Fluo4 time lapse Calcium imaging (white) at DIV70 superimposed with all neurons that were spontaneously active during imaging period of 60 s (1pseudo-colored in red, arrowheads). The image is produced from Methods video S4. Scale bar 100 μm. Insets on the right show activity of single neuron in dashed frame at 3 timepoints. (B) IF stainings showing long-term survival of neurons and increasing density of axonal networks positive for Tau in cultures on DIV70 (top), DIV 180 (middle) and DIV320 (bottom). Scale bar 50 μm. (C) Cortical neurons at DIV320 show putative dendritic spine-like protrusions. Scale bar 10 μm.

We further confirmed electrophysiological excitatory activity of the neurons and demonstrated evoked action potentials and typical resting membrane potential.^1^ Using Fluo4 time lapse Calcium imaging, we confirmed spontaneous neuronal activity of the majority of neurons in the cultures (Figure 5A and Methods video S4).

Methods video S4. Fluo4 time lapse Calcium imaging demonstrating abundant neuronal activity, expected outcomes section

Finally, the cortical neuron cultures obtained with this protocol can be cultured up to at least 12 months with the neuritic network becoming much denser over time, showing presence of putative dendritic-spine-like structures (Figures 5B and 5C).

## Limitations

In general, it is not possible to generate completely pure neuron cultures without any contaminating astrocytes. This is a well-known phenomenon, as both are derived from the same precursor cells.^8^^,^^12^ By using DAPT/5FU treatment and ensuring that single neurons are plated, the number of contaminating astrocytes can however be limited to a minimum using the presented protocol.

Details of the protocol need to be adjusted to the iPSC line in use. Potentially, the starting cell number as well as split ratios during differentiation need to be increased or decreased to adjust for different proliferative behavior of different cell lines. In our experience, the vast majority of cell lines successfully differentiate into cortical neurons with the provided cell numbers.

Furthermore, drying of the POL plates for making spots depends on the humidity of the environment. We suspect that it might be more difficult to dry the plates in very humid environments (>70% humidity). As a possible solution, we suggest using Silica beads close to the drying plate to reduce humidity. We did, however, not test this approach as this protocol was not developed or tested in a humid climate.

## Troubleshooting

### Problem 1

Aberrant differentiation of iPSCs prior to starting neuronal differentiation (step 3).

### Potential solution

• Suboptimal iPSC culture protocol and/or issues with media components. Optimize iPSC handling according to commonly used practice and replace all media and consumables to troubleshoot issue.
• Scratch off differentiated cells under sterile conditions, e.g., using a hood equipped with a dissection microscope.
• Decrease time of PBS-EDTA incubation, e.g., to 4 min; most pluripotent cells will still detach while aberrantly differentiated cells will stay attached to the plate when rinsing with media.

### Problem 2

Cells are not confluent or too dense after split into 12 well plate (step 3).

### Potential solution

• If cell density is too high, resulting in cell detachment, plate less cells; this is particularly important for very proliferative iPSC lines.
• If cell density is too low, resulting in holes in the cell layer, increase the number of plated cells during optimizations.
• If visible clumping occurs in the days after plating into 12-wells, increase incubation time with Accutase and ensure cells are triturated to single cells before plating.

### Problem 3

The cell line in use is difficult to differentiate into neuronal cells.

### Potential solution

In general, the presented differentiation protocol may need to be optimized and adjusted for each new iPSC line. In particular, the different proliferation rates of different cell lines need to be taken into consideration regarding cell number and timing of different splits as described in the text. Beyond that, another option for difficult-to-differentiate cells is to adjust the neuronal induction step to minimize stress for the cells due to the sudden change from E8 to induction medium.

The neuronal induction step can be adjusted as follows.

• use E6 – NI gradient on DIV0 and DIV1 starting with the split as described after step 3.
• On DIV0 perform split using 1 mL of E6-NI gradient medium-1 instead of NI.
• On DIV1 change medium E6-NI gradient medium-2.
• On DIV2 change medium to NI and use NI for all further feeds.

### Problem 4

Cells do not attach after making spots or cell density becomes lower over time.

### Potential solution

• Quality of the cells before making the spots is crucial.
  ○ Aberrant differentiation of iPSCs in the stem cell stage will impair the quality of the spots; it is therefore crucial to minimize aberrant differentiation before starting the differentiation to <5% as described in the “Before You Begin”-section.
  ○ It is important that the cell layer is homogeneous after induction: the cells should form a uniform layer without gaps and consistent density throughout the culture while on the 12-well plate,
  ○ As described in step 4, it is important to ensure high nutrient and media supply, in particular towards the last days of induction in the 12-well plate; cells need to be fed daily with sufficient media. Cells should not peel off at the rim of the well or start dying. If this is the case repeatedly, cell number should be adjusted, and spots should be made earlier (i.e., DIV5 or DIV6).
  ○ If dead cells are present or clumps form after collecting the cells from the 12-well plate, the spots and thus the entire differentiation are much more likely to fail.
• Low-quality drying of the POL-plates strongly affects attachment of spots to the plate; it is crucial that a uniform crystalline sheet without gaps is formed; if plates dry very fast (i.e., sheet is formed in <5–10 min) the airflow towards the plates should be reduced during drying to achieve drying times of 15–25 min.
• As mentioned in the limitations section, very humid conditions interfere with drying of the plates. Potential solutions might include adjustment of the airflow towards the plates or using Silica beads close to the drying plate to reduce humidity below 60%.

### Problem 5

Spots become less dense over time or cells start detaching (step 11).

### Potential solution

• If spots become less dense, bFGF can be added to NM medium for the first feeds after NI (so starting from DIV10) to ensure the density of cells in the spot stays high. However, this should be limited to 2–3 consecutive days as otherwise other neuronal fates or non-neuronal cells may arise.

### Problem 6

Cells are too sparse after Accutase splitting and/or rosettes were broken up during split (step 19).

### Potential solution

• NPCs should continue dividing after the split to re-form rosettes over the next days. This can be promoted by feeding with bFGF for 2 days as indicated in the protocol. If cells are still too sparse after 2 days, bFGF treatment can be continued for longer periods of time (e.g., until the next split).
• This may however also promote proliferation of aberrantly differentiated cells that were generated while the culture was not dense enough. We therefore recommend pausing bFGF treatment for at least 2 days between splits to promote cell death of aberrantly differentiated cells that may depend on bFGF signaling.

### Problem 7

Neuronal differentiations produce not only cortical forebrain, but also other neuronal cell types.

### Potential solution

• 5 μM XAV939, a WNT inhibitor, can be used on DIV0–2 (step 3/4) to promote cortical forebrain fate of the neurons.^13^

## Resource availability

### Lead contact

Further information and requests for resources and reagents should be directed to and will be fulfilled by the lead contact, Dominik Paquet (Dominik.Paquet@med.uni-muenchen.de).

### Materials availability

This study did not generate new unique reagents.

### Data and code availability

This study did not generate or analyze large datasets or code. Raw data for qPCR analysis is available from the corresponding author on request. The published article includes all other data generated or analyzed during this study.
